# Role of CA 19.9 in the Management of Resectable Pancreatic Cancer: State of the Art and Future Perspectives

**DOI:** 10.3390/biomedicines10092091

**Published:** 2022-08-26

**Authors:** Alessandro Coppola, Vincenzo La Vaccara, Tommaso Farolfi, Michele Fiore, Roberto Cammarata, Sara Ramella, Roberto Coppola, Damiano Caputo

**Affiliations:** 1 General Surgery, Fondazione Policlinico Universitario Campus Bio-Medico, 00128 Rome, Italy; 2 General Surgery, Università Campus Bio-Medico di Roma, 00128 Rome, Italy; 3 Radiation Oncology, Campus Bio-Medico University, 00128 Rome, Italy

**Keywords:** pancreatic ductal adenocarcinoma, CA 19.9, neoadjuvant treatment, upfront surgery, early detection, resectable pancreatic ductal adenocarcinoma

## Abstract

Background: Surgery still represents the gold standard of treatment for resectable pancreatic ductal adenocarcinoma (PDAC). Neoadjuvant treatments (NAT), currently proposed for borderline and locally advanced PDACs, are gaining momentum even in resectable tumors due to the recent interesting concept of “biological resectability”. In this scenario, CA 19.9 is having increasing importance in preoperative staging and in the choice of therapeutic strategies. We aimed to assess the state of the art and to highlight the future perspectives of CA 19.9 use in the management of patients with resectable pancreatic cancer. Methods: A PubMed database search of articles published up to December 2021 has been carried out. Results: Elevated pre-operative levels of CA 19.9 have been associated with reduced overall survival, nodal involvement, and margin status positivity after surgery. These abilities of CA 19.9 increase when combined with radiological or different biological criteria. Unfortunately, due to strong limitations of previously published articles, CA 19.9 alone cannot be yet considered as a key player in resectable pancreatic cancer patient management. Conclusion: The potential of CA 19.9 must be fully explored in order to standardize its role in the “biological staging” of patients with resectable pancreatic cancer.

## 1. Introduction

Pancreatic ductal adenocarcinoma [PDAC] represents one of the most lethal cancers, with poor survival rates even after surgical resection [1]. To date, this malignancy represents the fourth leading cause of cancer-related death, and due to its increasing incidence, it has been estimated to become the second one in the next future [2].

In the United States, accounting for 3% of all types of tumors, pancreatic cancer causes an annual average rate of deaths of 10.9 per 100,000 people [3]. This rate could be explained by PDAC biological aggressive behavior and by the lack of reliable tools for its early diagnosis [4].

Surgical resection represents the core of pancreatic cancer treatment [5] but, unfortunately, at the time of diagnosis, only 15–20% of patients are detected with early-stage PDAC and are eligible for resection [1,6]. Regarding the eligibility for surgery, on the bases of the results obtained with current clinical staging tools (e.g., CT scan, and MRI), four types of PDAC can be identified: resectable, borderline resectable, locally advanced, and metastatic [7]. Regrettably, up to 20–30% of radiological resectable PDACs show occult metastases at the moment of surgical exploration. Furthermore, most of the resected patients die due to early local or distant recurrence [8].

In recent years, the International Study Group of Pancreatic Surgery (ISGPS) and the International Association of Pancreatology (IAP) introduced the concept of biological resectability. Since elevated preoperative serum levels of carbohydrate antigen 19.9 (CA 19.9) have been associated with both the intraoperative detection of occult metastases and worse disease-free survival, even in resectable PDAC patients [9], CA 19.9 has become the main biological parameter to be used to assess biological resectability [7,10].

On this basis, Takahashi, in 2020, reported how preoperative CA 19.9 levels > 120 U/mL would allow one to define as “biologically” borderline resectable even tumors radiologically classified as resectable [11]. Therefore, the role of CA 19.9, already known and defined for diagnostic and prognostic purpose [12], has also acquired a preoperative staging role.

The CA 19.9 or Sialyl Lewis (a) antigen is derived from an aberrant pathway during the production of its normal unmeasured counterpart: the disialyl Lewis-a.

Up to 10% of the population is CA 19.9 non-secretor, with undetectable serum levels of this marker [13].

Being involved in the maintenance of immunological homeostasis and acting as a ligand for E-selectin on the endothelial cells, tumors secreting this molecule usually show biological aggressiveness [14].

Actually, non-secretor PDAC patients have better prognosis compared to CA 19.9 secretors [15].

However, in presence of PDAC, non-secretors present unmeasurable serum levels of CA 19.9 in only 41.9% of cases, while 27.4% still show levels of this marker > 37 U/mL [16].

Bergquist et al. reported that CA 19.9 may confer increased metastatic risk to resectable PDAC, with a consequent detriment in overall survival after surgical resection [17].

Thus, in recent years, many authors underlined the association between elevated pre-operative CA 19.9 levels and nodal involvement or positive margins at the pathological staging [18,19].

Hence, according to the above-mentioned PDAC behavior and to the encouraging results obtained with neoadjuvant treatments (NAT) in patients with borderline resectable and locally advanced disease, some authors suggested NAT even in patients with resectable PDAC instead of upfront surgery [20].

In 2019, Japanese researchers showed promising results on the adoption of NAT in resectable PDAC, with high overall survival rates after gemcitabine-based neoadjuvant chemotherapy compared with upfront surgery without any difference in terms of resection rate, margin status, and morbidity [21].

On these bases, in the era of multimodal treatment strategies, it is critical to understand the role that CA 19.9 may have on the choice of the more appropriate and advantageous therapeutic sequence. Here, we aimed to assess the state of the art and to highlight the future perspectives of CA 19.9 use in the management of patients with resectable PDAC. The association of preoperative CA 19.9 with other biomarkers, radiological findings, pathological nodal involvement (N+), the margin status positivity (R+) need of vascular resection (VR), and early local recurrence (ELR) after surgery have been particularly focused on the association with postoperative overall survival (OS).

## 2. Materials and Methods

A PubMed database search of articles published up to December 2021 has been carried out. Different combinations of the following terms have been used: pancreatic cancer, pancreatic adenocarcinoma, CA 19.9, resectable pancreatic adenocarcinoma, borderline resectable pancreatic carcinoma, neoadjuvant treatments, nodal involvement, margin status, and overall survival. Only articles published in English with available full text have been considered without limitation concerning article types (original articles, review, etc.). References reported in the selected articles have been also considered as other bibliographic sources. According to the above-mentioned criteria, 47 papers have been considered.

## 3. Results

### 3.1. CA 19.9 and Nodal Involvment

The Heidelberg group underlined how N+ still represents the most relevant prognostic factor for long-term oncological outcomes of patients affected by pancreatic cancer who underwent upfront surgical resection [17,22].

Additionally, the American Joint Committee on Cancer (AJCC) highlighted the role that lymph-node invasion has on patients’ prognosis. In the latest edition of the TNM staging system for PDAC, the N category has been modified and patients with metastases in four or more lymph-nodes (i.e., pN2) switched from stage IIB to stage III [23].

Therefore, considering the prognostic role of N+ in PDAC underwent surgical resection, we can assume that the preoperative assessment of lymph node status would be crucial for the choice between neoadjuvant treatments and upfront surgery in resectable PDACs.

Unfortunately, tools routinely used for the radiological staging of PDAC (e.g., CT scan, MRI, etc.) are impaired by a not negligible rate of down-staged tumors; notably, a lack of sensitivity in nodal involvement assessment is usually reported [24].

Hence, CA 19.9 and other markers have been proposed to improve the accuracy of radiological staging.

In 2010, Nanashima and colleagues analyzed a series of 139 patients with biliary and pancreatic carcinomas who underwent surgical resection. The authors reported a significant improvement in the accuracy of the assessment of nodal involvement when CA 19.9 and radiological findings were combined. Notably, the higher positive predictive value (PPV) and sensitivity were observed for nodal metastases in the hepatoduodenal ligament [25].

Mattiucci et al. in 2019, in a large multicenter study (more than 700 PDACs), showed a significant association between CA 19.9 and N+ (*p* < 0.001) [26].

In 2021, Mattiucci’s findings have been confirmed in a retrospective study including a series of 165 patients. In this experience, increased preoperative levels of CA 19.9 were significantly associated with N+. Notably, this association was not confirmed in patients with hypoalbuminemia [19].

In a retrospective study including 160 patients who underwent surgical resection, Wang and colleagues tried to define a standard method able to identify N+ before surgery.

They staged tumors using the combination of serum CA 19.9 levels and positron emission tomography-computed tomography (PET/CT) scan findings. The identified SUV values and CA 19.9 cut-offs able to predict lymph nodes micro-metastases were 7.05 (sensitivity 71.2%; specificity 76.6%) and 240.55 U/mL (sensitivity 62.1%; specificity 79.8%), respectively [27].

Supported by these results, using a multivariate logistic regression model, Hua elaborated a nomogram able to predict N+ after PD in a series of 558 resected PDAC.

The nomogram was based on the combination of a risk score calculated on the pre-operatory serum value of four markers (CA 19.9 with Ca 125, Ca 50, and Ca 242) and radiological staging findings with particular regards to the N status.

The obtained prediction model demonstrated good ability to distinguish between patients at lower or higher risk of lymph-nodes metastases at pathological staging [28].

Table 1 reports the main characteristics and findings of the above-mentioned studies.

### 3.2. CA 19.9 and Margin Status

The impact that negative resection margins (R0) after surgery have in PDAC prognosis is well known [19].

Moreover, the importance to refer to the Leeds Pathology Protocol (LEEPP) to assess the R1 status is recognized [29].

Regarding the role of CA 19.9 in predicting R+, in 2018 Lai et al. published a study including 189 patients with resected PDAC in a time span of ten years and investigated different pre-operatory factors able to predict R+. They demonstrated that elevated levels of CA 19.9 (cut-off 246 U/mL) were an independent risk factor for R+ (OR = 2.318; *p* = 0.040). Additionally, the presence of tumor in the uncinate process (OR = 2.996; *p* = 0.015) was an independent risk factor for R+ [30].

These findings were not confirmed by the study of Moschera and colleagues in 2019. In a series including 184 patients, they studied the potential association between CA 19.9 and R+; the study population was divided according to various preoperative CA 19.9 levels. Unlike others, they did not find any association with R+, even when CA 19.9 > 1000 U/mL was considered [31].

In 2020, Fiore and colleagues, considering that R+ is one of the most important predictors of early recurrence after PD, demonstrated a significant association between CA 19.9 levels and R+. In a series of 120 patients, they analyzed the pre-surgical CA 19.9 level, the maximum SUV, the metabolic tumor volume (MTV), and total lesion glycolysis (TLG) at 18F-FDG PET/CT. Using ROC curve analysis to assess the cut-offs for all those variables, they found a significant association between pre-surgical CA 19.9 levels, OS, and early recurrence. Notably, they reported a six-time higher risk of early recurrence in the presence of a preoperative level of CA 19.9 > 698 U/mL [32].

More recently, Coppola et al. also demonstrated that high levels of preoperative CA 19.9 might predict R+. More specifically, they showed that serum levels of CA 19.9 > 730 U/mL were significantly observed in R+ (specificity 85%, despite low PPV 63% and NPV 66%). Moreover, decreasing the CA 19.9 cut-off level to 418 U/mL, they obtained a similar specificity (87%) [19].

In 2021, Bergquist and colleagues at the Mayo Clinic also confirmed these data. In a retrospective review including more than 12,000 patients, they showed that more than 60% of R+ had elevated preoperative serum levels of CA 19.9 [33].

Table 2 reports the main characteristics and findings of the above-mentioned studies.

### 3.3. CA 19.9 and Vascular Invasion

The association between pre-operative CA 19.9 serum levels and intraoperative finding of vascular invasion, whether venous or arterial, is unclear.

In 2006, Ferrone et al., in a study including 176 resectable PDACs who underwent surgery, reported that preoperative CA 19.9 levels can predict stage and vascular invasion [34].

Few years later, Barton and colleagues, reviewing 143 patients undergoing pancreatoduodenectomy for pancreatic adenocarcinoma, did not find any significant association between CA 19.9 levels and vascular involvement. More specifically, preoperative CA 19.9 serum levels, even if higher than 120 U/mL, were not associated with increased rates of R1 and R2 resection (*p* = 0.86), the involvement of the superior mesenteric artery (SMA) (*p* = 0.88), or the involvement of the portal vein groove (*p* = 0.14) [9].

Pandiaraja lately confirmed these findings in a prospective study including 30 consecutive patients. However, due to the very small number of patients analyzed, the reported conclusions must be carefully taken into consideration [35].

In 2019, Kowalchuk et al. published the results of a retrospective study, including more than five hundred patients, hypothesizing that elevated CA 19.9 levels would be associated with worse pathologic findings and oncologic outcomes. Using CA 19.9 levels at the cut-off value of 55 U/mL, they found a significant association between this marker and the pT stage. More specifically, CA 19.9 levels higher than the cut-off resulted associated with pT stage ≥ 3 (*p* = 0.0005) [36].

Additionally, Coppola et al. demonstrated a statistically significant association between CA 19.9 and T-stage (*p* = 0.02) and higher rates of vascular resection (*p* = 0.03) [19].

Table 3 reports the main characteristics and findings of the above-mentioned studies.

### 3.4. CA 19.9 and Early Local Recurrence

Another relevant feature of PDAC is the tendency towards ELR, defined as recurrence within six months from the resection. In this section, studies reporting data about the association between preoperative serum levels of CA 19.9 and the risk of early local recurrence after resection have been analyzed.

In 2007, Kang et al. retrospectively studied a group of 61 patients and found that CA 19.9 levels, adjusted according to bilirubin levels, at the cut-off of 50 U/mL, using a multivariate analysis, were an independent predictive factor of recurrence after pancreatic cancer curative resection (*p* = 0.027) [37].

Ten years later, Nishio confirmed those findings in a study that aimed to identify the preoperative clinicopathological features of early recurrence after curative resection of PDAC. In a series of 90 patients, preoperative serum CA 19.9 levels ≥ 529 U/mL were significantly associated with recurrence within 1 year after surgery at a univariate analysis (*p* = 0.0011). Multivariate analysis confirmed that CA 19.9 was an independent risk factor for ELR [38].

Based on those findings, Kurahara et al. published a paper confirming the significant association between serum levels of CA 19.9 > 85 U/mL and ELR.

The authors suggested NAT for patients at higher risk of ELR, in order to improve their prognosis [39].

In 2019, Suzuki and colleagues retrospectively analyzed a study group of 149 patients and assessed a prognostic score that combines distance from common hepatic artery (CHA) or superior mesenteric artery (SMA) and preoperative serum levels of CA 19.9 able to predict local recurrence in patients with pancreatic cancer after surgery [40]. Table 4 reports the main characteristics and findings of the above-mentioned studies.

### 3.5. CA 19.9 and Overall Survival

Preoperative CA 19.9 levels have been reported to be a reliable tool to predict the overall survival of PDAC patients. As reported by Ferrone et al. in 2006, patients with preoperative CA 19.9 levels < 1000 U/mL median survival rates were higher than those of patients with CA 19.9 levels > 1000 U/mL (28 months vs. 12 months) [34].

Few years later, Barton and colleagues reported, in a series of 143 patients who underwent pancreatoduodenectomy for PDAC, increased OS and disease-free survival in presence of preoperative serum values of CA 19.9 lower than 120 U/mL [9].

In 2012, Ballehaninna and colleagues confirmed these results. In their review, patients with normal presurgical serum levels of CA 19.9 (<37 U/mL) had longer median survival (32–36 vs. 12–15 months). They also highlighted the prognostic role of post-treatment CA 19.9, showing that the normalization or a significant decrease (≥20–50% from baseline) of marker levels after NAT was associated to better survival [41].

Based on these findings, Yang et al., in 2013, also evaluated the importance of a CA 19.9 decrease after NAT, demonstrating that patients with CA 19.9 reduction > 90% had improved OS (16.2 vs. 7.5 months). In their work, they also showed that the median OS of patients with CA 19.9 levels <85.5 U/mL after NAT was of 10.3 months, compared to 7.1 months of patients with higher levels of the marker [42].

In 2014, Dong et al. published a retrospective study of 120 resectable PDAC who underwent surgery and confirmed that preoperative elevated CA 19.9 levels (optimal cut-off value of 338.45 U/mL) were an independent predictor of poor prognosis in PDAC [18].

A further step was taken in 2019 by Mattiucci et al. In their work, they divided the population study based on pre-surgical CA 19.9 levels in four groups: 5–37, 37–100, 100–353, and >353 U/mL, showing that the subgroup of patients with preoperative CA 19.9 serum levels between 100 and 353 and >353 U/mL had worse overall survival and disease-free survival [26].

Nowadays, as highlighted by Ye and colleagues, post-neoadjuvant treatment CA 19.9 variation represents an important prognostic factor for OS of PDAC patients.

In their systematic review, CA 19.9 reduction >50% or a complete normalization of CA 19.9 after NAT was significantly associated with better overall survival (*p* < 0.001), suggesting that CA 19.9 level response may represent a prognostic index useful for therapeutic decisions [43].

In agreement with what previously reported, recently Takahashi et al., in a retrospective study including 407 patients, reported that resectable PDACs with preoperative CA 19.9 levels ≥ of 120 U/mL had OS similar to patients with borderline resectable tumors. Furthermore, in their work they also demonstrated that the normalization of CA 19.9 levels after NAT represents a significant positive prognostic factor [11].

Table 5 reports the main characteristics and findings of the above-mentioned studies.

## 4. Discussion

To date, CA 19.9 is the only Food and Drugs Administration (FDA)-approved marker in clinical practice for pancreatic cancer. However, due to its low specificity (82%) and sensitivity (79%), it cannot be recommended for the diagnosis of PDAC but has been confirmed to be useful in the follow-up after surgery and systemic treatments.

Over the past twenty years, many researchers have been investigating other potential applications of CA 19.9 in PDAC management. Associations with different stages of the disease and with the response to different types of treatments were particularly evaluated. Unfortunately, often contradictory results have been reported, and in any case, univocal cut-offs have never been identified.

Moreover, the era of multimodal treatments gave a boost to the exploitation of the potential ability of CA 19.9 in predicting the response to treatment, in order to avoid unnecessary toxicity and useless invasive surgical procedures.

In this scenario, CA 19.9 gained a crucial role in PDAC preoperative staging and may represent a user-friendly and cheap tool to establish the more appropriate therapeutic option. This evidence leads to the recent concept of the biological staging of PDAC.

Regarding the ability of CA 19.9 to predict the positivity of the lymph node status before surgery, the risk of a resection with positive margins, the need to proceed with a vascular resection, and the analysis of the literature we conducted ( which certainly is not to be considered exhaustive) would seem to confirm the potential of CA 19.9 [9,17,19,25,26,27,28,29,30,31,32,33,34,35,36].

As a result, CA 19.9 could be a candidate to play a key role in the management of patients affected by resectable PDAC, too.

More specifically, elevated levels of CA 19.9 have been associated with more advanced stages of the disease, and according to the recent definition of “biological” unresectable PDAC, tumors are classified as resectable; according to the conventional radiological staging tools results, they should be considered borderline resectable in presence of elevated levels of CA 19.9.

On this basis, and taking into account the promising results obtained with NAT in borderline resectable PDACs, patients with resectable PDACs and elevated levels of CA 19.9 could be candidate for NAT.

Moreover, the persistence of elevated levels of CA 19.9 after NAT could identify patients with biologically aggressive tumors that could not benefit from surgical resection, even if technically feasible, since the postoperative risks may overcome the oncological benefits.

Two randomized trials have recently focused on this theme: the Dutch Randomized Phase III PREOPANC Trial [20] and the Japanese Randomized phase II/III trial (Prep-02/JSAP05) one [21].

While the Japanese trial reported interesting and promising results on the adoption of NAT, which could allow for increased OS without differences in terms of the resection rate, margin status, and morbidity, the Dutch experience did not find any OS benefit in the NAT group compared to the upfront surgery group, even if increased rates of R0, N0, and disease-free survival have been found.

On the other hand, however, most of the literature we analyzed reports encouraging data about the association of CA 19.9 levels with the onset of early local recurrence and with overall survival [9,11,18,26,37,38,39,40,41,42,43].

Unfortunately, to date, all the data reported in the literature are affected by strong limitations that do not allow generalize those findings. The first limitation is that most of the published studies are retrospective and monocentric and have been often conducted on a small series.

Second, there is no agreement regarding the timing of serum CA 19.9 dosage. If assessed too early from surgery, its reliability could be impaired by cancer progression.

Third, other confounding factors that could impair the role of CA 19.9 (such as the presence of cholangitis and jaundice of hypoalbuminemia) have often not been considered.

Fourth, as previously reported, up to 10% of the PDAC patients do not secrete CA 19.9. Furthermore, CA 19.9 secretion can be strongly influenced even by tumor burden and cellular secretion rate [14,44,45].

On this basis, it is evident that further prospective multicenter studies on larger and more homogenous series are needed to better define the role of CA 19.9 in PDAC management.

Nonetheless, it has to be considered that interesting perspectives are emerging, due to recent advances in multiomics, that may lead to the discovery of other biomarkers using genomics, transcriptomics, proteomics, metabolomics, glycomics, and metagenomics. In this scenario, panels of markers combining CA 19.9 with other novel biomarkers from different “omics” levels are showing promising results in pancreatic cancer early detection [46] and in advancing its precision management [47].

## 5. Conclusions

Most of the study published up to date, even though with different not negligible limitations, demonstrated that CA 19.9 may be an effective predictor of pathological nodal involvement, vascular invasion, and margin status positivity after PDAC radical surgery. Nonetheless, CA 19.9 levels are strictly associated with a risk of ELR and OS.

Further investigations are needed to better define the role of CA 19.9 in pancreatic cancer management overall, considering that it is mandatory to improve the preoperative staging of PDAC patients.

Radiological staging has to be combined with “biological staging”, and more accurate tools in these fields represent urgent tasks.

With the aim to aid in tackling this issue, new technologies in the field of “omics” are showing promising results that can contribute to the identification of new biomarkers able to improve the utility of CA 19.9.

## Figures and Tables

**Table 1:** Main characteristics and findings of studies reporting data about association between CA 19.9 levels and nodal involvement.

Ref. n°	Author	Year	N° of Patients	Type	Concept Resumed
[17]	Bergquist	2016	10,806	Systematic review	Patients with elevated CA 19.9 levels (>37 U/mL) were more likely to have N1 disease (*p* < 0.001). This result was confirmed both for modest or greater elevation. Early staged PDACs with elevated CA 19.9 had decreased survival at 1, 2, and 3 years (56% vs. 68%, 30% vs. 42%, and 15% vs. 25%, all *p* < 0.001.
[25]	Nanashima	2010	139	Retrospective	More accurate preoperative assessment of nodal involvement using combination of CA 19.9 and radiological findings at the CT scan (*p* = 0.003).
[26]	Mattiucci	2019	700	Retrospective	Higher rates of N+ in patients with higher preoperative CA 19.9 level (*p* < 0.001). Worse overall survival (OS) and disease-free survival in patients with preoperative CA 19.9 serum levels between 100 and 353 and >353 U/ml.
[19]	Coppola	2021	165	Retrospective	Increased preoperative CA 19.9 levels significantly associated with N+ in presence of normal serum level of albumin (*p* < 0.001).
[27]	Wang	2019	160	Retrospective	Combination of CA 19.9 and PET/CT scan findings showed promising results in identifying patients at higher risk of N+. Cut-offs used were CA 19.9 levels > 240.55 U/mL and SUV of 7.05 (*p* < 0.001).
[28]	Hua	2021	558	Retrospective	Combination of CA 19.9 with Ca 125, Ca 50, and Ca 242 and radiological findings are used to develop a nomogram to predict the risk of pathological nodal involvement (*p* = 0.0009).

**Table 2:** Main characteristics and findings of studies reporting data about association between CA 19.9 levels and margin status.

Ref. n°	Author	Year	N° of Patients	Type	Concept Resumed
[29]	Nappo	2021	168	Retrospective	Importance to refer to the Leeds Pathology Protocol (LEEPP) to assess the R1 status (*p* < 0.05).
[30]	Lai	2018	189	Retrospective	Elevated levels of CA 19.9 result in a two-time higher risk of R+ resection (*p* = 0.040). R+ had no impact on OS.
[31]	Moschera	2019	184	Retrospective	Even very high (>1000 U/mL) preoperative levels of CA 19.9 were not associated with increased risk of R+ resection, (*p* < 0.05).
[32]	Fiore	2020	120	Retrospective	Levels of CA 19.9 > 698 U/mL were associated to a six-time higher risk of R+ (*p* = 0.005).
[19]	Coppola	2021	165	Retrospective	In presence of normo-albuminemia, serum levels of CA 19.9 > 730 U/mL were significantly associated to R+ (specificity 85%, despite low PPV 63% and NPV 66%) (*p* = 0.025).
[33]	Bergquist	2021	12082	Review	More than 60% of R+ had elevated preoperative serum levels of CA 19.9 (*p* < 0.001).

**Table 3:** Main characteristics and findings of studies reporting data about association between CA 19.9 levels and vascular invasion.

Ref. n°	Author	Year	N° of Patients	Type	Concept Resumed
[34]	Ferrone	2006	176	Retrospective	Preoperative CA 19.9 levels can predict vascular invasion in patient with resectable pancreatic adenocarcinoma (*p* = 0.03).
[9]	Barton	2009	143	Retrospective	Preoperative CA 19.9 serum level is not associated with a vascular invasion. Patients with CA 19.9 lower than 120 U/mL had an increased OS and disease-free survival (*p* = 0.002).
[35]	Pandiaraja	2016	30	Prospective	A high preoperative level of CA 19.9 was not related with an increased risk of vascular resection.
[36]	Kowalchuk	2019	509	Retrospective	Preoperative CA 19.9 levels predict vascular invasion (*p* = 0.0072) but are not associated with poorer OS (HR = 1.6 (1.3–2.0)).
[19]	Coppola	2021	165	Retrospective	CA 19.9 levels were significantly associated with higher rates of vascular resection (*p* = 0.03).

**Table 4:** Main characteristics and findings of studies reporting data about association between CA 19.9 levels and early local recurrence.

Ref. n°	Author	Year	N° of Patients	Type	Concept Resumed
[37]	Kang	2007	60	Retrospective	CA 19.9 levels (cut off ≥ 50 U/mL) adjusted according to bilirubin levels were an independent predictive factor of recurrence after curative resection of pancreatic cancer (*p* = 0.027).
[38]	Nishio	2017	90	Retrospective	Preoperative CA 19.9 ≥ 529 U/mL was an independent risk factor for early local recurrence
[39]	Kurahara	2018	115	Retrospective	Serum levels of CA 19.9 level > 85 U/mL were significantly associated with early local recurrence after surgery (*p* = 0.028).
[40]	Suzuki	2019	149	Retrospective	Distance from common hepatic artery (CHA) and superior mesenteric artery (SMA), and preoperative serum levels of CA 19.9, predict local recurrence in resected PDAC patients (*p* = 0.004).

PDAC: pancreatic ductal adenocarcinoma.

**Table 5:** Literature review on relationship between CA 19.9 and overall survival (OS).

Ref. n°	Author	Year	N° of Patients	Type	Concept Resumed
[34]	Ferrone	2006	176	Retrospective	Patients with a CA 19.9 < 1000 U/mL had higher median survival rates compared to those with CA 19.9 > 1000 U/mL (28 months vs. 12 month) (*p* = 0.0005)
[9]	Barton	2009	143	Retrospective	Patients with CA 19.9 lower than 120 U/mL had increased overall survival and disease-free survival rates (*p* = 0.002).
[41]	Ballehaninna	2012	90	Review	Normal serum level of presurgical CA 19.9 (<37 U/mL) means longer median survival (32–36 months) compared to patients with levels >37 U/mL (12–15 months).
[42]	Yang	2013	115	Retrospective	After NAT, CA 19.9 reduction > 90% allows better overall survival (16.2 vs. 7.5 months) (*p* = 0.01).
[18]	Dong	2014	120	Retrospective	Elevated preoperative CA 19.9 levels were independent predictive factor of poor prognosis in PDAC patients (optimal cut-off value of 338.45 U/mL) (*p* = 0.04).
[26]	Mattiucci	2019	700	Retrospective	Patients with preoperative CA 19.9 serum levels between 100 and 353 and >353 U/mL had worse overall survival and disease-free survival (*p* < 0.001).
[43]	Ye	2020	2242	Meta-Analysis	CA 19.9 decrease > 50% or a complete normalization of CA 19.9 after neoadjuvant treatment were significantly associated with better overall survival (*p* < 0.0001).
[11]	Takahashi	2020	407	Retrospective	Patients with radiologically resectable tumor but CA 19.9 > 120 U/mL had OS similar to patients with borderline resectable tumors (44% vs. 34%, *p* = 0.082). Normalization of CA 19.9 after neoadjuvant chemotherapy represents an important positive prognostic factor.

## Data Availability

Not applicable.
